# FOXM1 Inhibition Enhances the Therapeutic Outcome of Lung Cancer Immunotherapy by Modulating PD‐L1 Expression and Cell Proliferation

**DOI:** 10.1002/advs.202202702

**Published:** 2022-08-17

**Authors:** Hamadi Madhi, Jeon‐Soo Lee, Young Eun Choi, Yan Li, Myoung Hee Kim, Yongdoo Choi, Sung‐Ho Goh

**Affiliations:** ^1^ Research Institute National Cancer Center 323 Ilsan‐ro, Goyang Gyeonggi‐Do 10408 Republic of Korea; ^2^ Department of Anatomy Graduate School of Medical Sciences Yonsei University College of Medicine Seoul 03722 Republic of Korea

**Keywords:** forkhead box protein M1, immunotherapy, lung cancer, programmed death‐ligand 1, thiostrepton

## Abstract

Programmed death‐ligand 1 (PD‐L1) is a major target to cancer immunotherapy, and anti‐PD‐L1 and anti‐PD‐1 antibody‐mediated immunotherapy are being increasingly used. However, immune checkpoint inhibitors (ICIs) are ineffective in treating large tumors and cause various immune‐related adverse events in nontarget organs, including life‐threatening cardiotoxicity. Therefore, the development of new therapeutic strategies to overcome these limitations is crucial. The focus of this study is the forkhead box protein M1 (FOXM1), which is identified as a potential therapeutic target for cancer immunotherapy and is associated with the modulation of PD‐L1 expression. Selective small interfering RNA knockdown of FOXM1 or treatment with thiostrepton (TST) significantly reduces PD‐L1 expression in non‐small‐cell lung cancer (NSCLC) cells and inhibits proliferation. Chromatin immunoprecipitation‐PCR reveals that FOXM1 selectively upregulates PD‐L1 expression by binding directly to the PD‐L1 promoter. In vivo animal studies have shown that TST treatment significantly downregulates PD‐L1 expression in human NSCLC tumors, while greatly reducing tumor size without side effects on normal tissues. Combined treatment with TST and anti‐4‐1BB antibody in the LLC‐1 syngeneic tumor model induces synergistic therapeutic outcomes against immune resistant lung tumors as well as 2.72‐folds higher CD3^+^ T cells in tumor tissues compared to that in the anti‐4‐1BB antibody treatment group.

## Graphical abstract

In this study, the authors show that FOXM1 selectively regulates PD‐L1 expression by binding directly to the PD‐L1 promoter. The FOXM1 inhibitor significantly lowers PD‐L1 levels and tumor growth in vitro and in vivo without any side effects on vital normal tissues. Combined therapy with a FOXM1 inhibitor and anti‐4‐1BB antibody synergistically enhances cancer immunotherapy of immune‐resistant lung tumors.

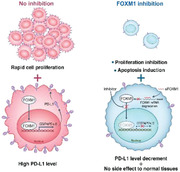

## 1 Introduction

Lung cancer is the most common form of cancer in the world. Despite tremendous therapeutic progress, non‐small‐cell lung cancer (NSCLC) still accounts for 80–85% of human lung cancer cases with high morbidity.^[^ ^1^ ^]^ Although immune cells infiltrate many NSCLC tissues, cytotoxic T‐lymphocytes in tumor tissues are functionally inhibited by various mechanisms employed by the tumor cells, including the aberrant upregulation of immune checkpoint proteins.^[^ ^2^ ^]^

Programmed death‐ligand‐1 (PD‐L1), is encoded by the CD274 gene and is a crucial immune checkpoint molecule that mediates the interaction between lung cancer cells and tumor‐infiltrating T cells. When used as monotherapy or in combination with other drugs, immune checkpoint inhibitors (ICIs), such as anti‐PD‐L1 or PD‐1 antibodies, have shown remarkable efficacy in lung cancer treatment by targeting the immune evasion mechanism of cancer cells.^[^ ^3^ ^]^ However, as the clinical applications of ICI treatments have increased, various immune‐related adverse events in non‐target organs, including life‐threatening cardiac complications, have been reported.^[^ ^4^, ^5^, ^6^ ^]^ The high cost of ICI treatment presents a huge burden on patients with cancer.^[^ ^7^ ^]^ Moreover, a previous study has indicated that the effect of ICI on tumor treatment was not significant when the tumor size was large, or the tumor growth rate was fast.^[^ ^8^ ^]^ Therefore, the development of new therapeutic drugs or strategies to inhibit both PD‐L1 expression and cell proliferation in NSCLC is imperative and remains an ongoing challenge.

Forkhead box protein M1 (FOXM1) is important for regulating various processes involved in lung cancer tumorigenesis, including cell cycle progression,^[^ ^9^ ^]^ cancer therapy resistance,^[^ ^10^ ^]^ and metastasis.^[^ ^11^ ^]^ These functions are attributed in part to the ability of FOXM1 to translocate to the nucleus and bind to the regulatory regions of several target genes, through their versatile forkhead or DNA‐binding domain, and are crucial for the survival of cancer cells.^[^ ^12^ ^]^ Compounds such as thiostrepton (TST), siomycin A,^[^ ^13^ ^]^ Robert Costa Memorial drug‐1 (RCM‐1),^[^ ^14^ ^]^ and Forkhead domain inhibitor‐6 (FDI‐6)^[^ ^15^ ^]^ have been identified as putative FOXM1 inhibitors. Among these, TST, a natural thiazole antibiotic isolated from *Streptomyces azureus*, inhibits the interaction of the forkhead domain of FOXM1 with the target DNA.^[^ ^16^ ^]^ TST has been used to treat mastitis caused by gram‐negative bacteria^[^ ^17^ ^]^ and is reported active against breast cancer.^[^ ^18^ ^]^

The present study demonstrates that FOXM1 selectively upregulates PD‐L1 expression by binding directly to the PD‐L1 promoter. In vitro experiments involving selective knockdown of FOXM1 mRNA using FOXM1‐specific small interfering RNA (siRNA; siFOXM1) or FOXM1 inhibition by TST significantly reduced PD‐L1 expression and proliferation of NSCLC cells. In vivo data further supported the hypothesis that FOXM1 is a promising target for inhibiting both PD‐L1 expression and cell proliferation in NSCLC. In particular, combined treatment of TST and anti‐4‐1BB antibody in LLC‐1 syngeneic tumor model induced synergistic therapeutic outcome against immune‐resistant lung tumors as well as 2.72‐folds increase in the CD3^+^ T cells in tumor tissues compared with that in the anti‐4‐1BB antibody treatment group.

## 2 Results

### 2.1 Relation of FOXM1 to PD‐L1 Expression in Lung Cancer Cells

**Figure 1:**
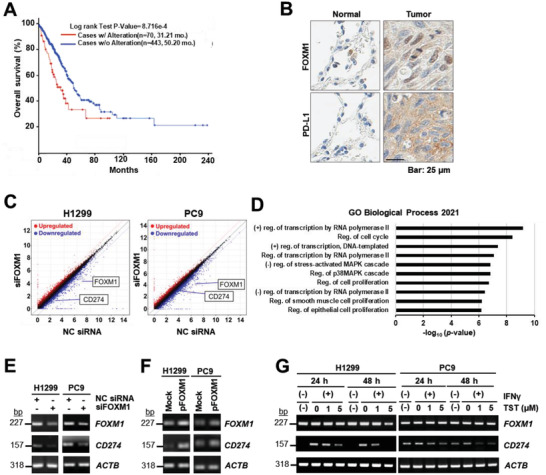
PD‐L1 is downregulated in FOXM1‐knockdown LUAD cell lines. A) Overall survival curves (*p* = 8.716 × 10^−4^) (*n* = 513) for patients with lung adenocarcinoma with (red line) or without (blue line) simultaneous alterations in expressions of both FOXM1 and PD‐L1 from TCGA Firehose LUAD dataset. B) Immunohistochemical analysis of expression levels of FOXM1, and PD‐L1 in human lung cancer tissues and normal lung tissues (scale bar; 25 µm). C) RNA‐seq analysis was performed in H1299 and PC9 cells transfected with siFOXM1 or non‐specific control (NC) siRNA. D) Gene ontology functional enrichment analysis showed enriched biological processes in 67 genes that were downregulated following siFOXM1 treatment in H1299 and PC9 cell lines for 72 h. The transcription level of FOXM1 and PD‐L1 following FOXM1 knockdown by E) siRNA for 72 h, or F) following transfection with FOXM1 isoform b (pFLAG‐FOXM1) or an empty vector (Mock) for 48 h or G) TST treatment at a concentration of 1 or 5 µm in the presence of IFN*γ* (20 ng mL^−1^) for 24 or 48 h were assessed by performing semi‐quantitative PCR. *β*‐actin was used as a loading control.

To explore the clinical relevance of FOXM1 and PD‐L1 in patients with adenocarcinoma, we analyzed the survival curves of lung adenocarcinoma (LUAD) in The Cancer Genome Atlas (TCGA) dataset on cBioPortal. Interestingly, 70 patients with higher FOXM1 and PD‐L1 expression exhibited lower median overall survival rate than the 443 patients without any alteration in FOXM1 and PD‐L1 expressions (31.21 vs 50.20 months, *p =* 8.716 × 10^−4^; **Figure** **1**A). We hypothesized that FOXM1 is a putative transcription factor (TF) that regulates PD‐L1 expression, and is associated with poor prognosis in patients with LUAD. We immunostained tumor tissues from lung cancer tissues with antibodies against FOXM1 and PD‐L1, and compared the expression of FOXM1 and PD‐L1 to their corresponding normal counterparts. Interestingly, FOXM1 and PD‐L1 were upregulated in LUAD tissues when compared to the adjacent normal tissues (Figure 1B).

RNA‐seq analysis was performed on H1299 and PC9 cells following siRNA‐mediated FOXM1 knockdown. Interestingly, among the 67 differentially expressed genes (DEGs) with a lower than 0.5‐fold expression in both cell lines (Table S1, Supporting Information), the CD274 gene encoding PD‐L1 was identified as the putative target of FOXM1 (Figure 1C). To determine the biological function of the downregulated genes following FOXM1 knockdown in H1299 and PC9 cells, we subjected the downregulated genes (67 in total) to gene set enrichment analysis (GSEA) using the Enrichr database.^[^ ^19^, ^20^, ^21^ ^]^ Positive transcriptional regulation by RNA polymerase II and cell cycle regulation were significantly enriched in the target genes (Figure 1D). This suggests that FOXM1 may also contribute to CD274 regulation. The encoded PD‐L1 protein is crucial for immune evasion by lung cancer cells. Therefore, we examined PD‐L1 expression after siRNA‐mediated FOXM1 knockdown. PD‐L1 transcription levels were observed to be significantly decreased in both cell lines following FOXM1 knockdown (Figure 1E). We further assessed PD‐L1 transcription levels following FOXM1 overexpression using pFLAG‐FOXM1 isoform b, which is the major isoform expressed under physiological conditions. Although FOXM1 knockdown resulted in a significant decrease in PD‐L1 transcription levels, FOXM1 overexpression resulted in a significant increase in PD‐L1 mRNA expression in H1299 and PC9 cells (Figure 1F). The potential effects of FOXM1 on PD‐L1 expression in NSCLC cells were assessed using FOXM1 inhibition strategies, other than siRNA knockdown. Several FOXM1 inhibitors including TST, a natural product that inhibits FOXM1, were tested in H1299 and PC9 cells (data not shown), and TST was the most efficient inhibitor and significantly reduced FOXM1 levels. We analyzed PD‐L1 transcription levels in cells treated with TST in the presence of IFN‐*γ* to elicit an increase in PD‐L1 expression. Interestingly, TST‐induced FOXM1 reduction triggered a concentration‐dependent reduction in PD‐L1 mRNA expression in both the cell lines (Figure 1G).

### 2.2 FOXM1 Repression Suppresses Cell Growth and Induces NSCLC Cell Apoptosis

**Figure 2:**
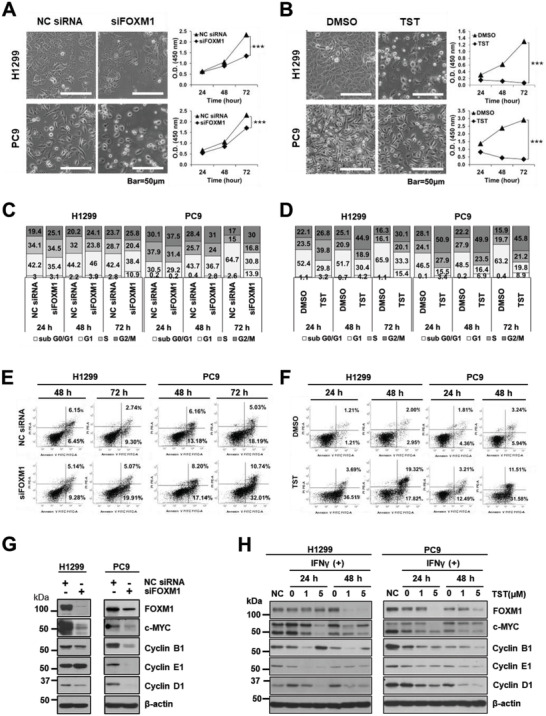
TST‐mediated downregulation of FOXM1 is associated with reduced cell proliferation, reduced survival, and increased apoptosis. H1299 and PC9 cells exhibiting apoptotic phenotypes upon FOXM1‐depletion using A) NC siRNA or siFOXM1 transfection for 72 h or B) undergoing DMSO or TST (5 µm) treatment for 48 h. Cells were imaged using a phase‐contrast microscope (scale bar, 50 µm). Cell proliferation was monitored by performing a WST‐1 assay at indicated time points. C) FACS analysis of DNA content by PI staining on H1299 or PC9 NC siRNA or siFOXM1 transfected cells for 24, 48, or 72 h or on D) DMSO or TST (5 µm) treated cells for 24 and 48 h. E) FACS analysis of FOXM1‐depleted H1299 or PC9 apoptotic cells stained with Annexin V and PI using NC siRNA or siFOXM1 transfection for 72 h or F) DMSO or TST (5 µm) treated cells for 24 and 48 h. All annexin V positive cells were counted as apoptotic cells and represented as a percentage of the total population. G) Protein expression of FOXM1, c‐MYC, cyclin B1, cyclin E1, and cyclin D1 in siFOXM1‐knockdown cells. *β*‐Actin was used as the loading control. The representative image is from duplicate experiments with comparable results. H) Protein expression of FOXM1, c‐MYC, cyclin B1, cyclin E1, and cyclin D1 in H1299 and PC9 cells treated with IFN*γ* (20 ng mL^−1^) and TST (1 or 5 µm) for 24 or 48 h. *β*‐actin was used as an internal control.

To confirm the effects of FOXM1 on cell proliferation and survival in NSCLC, time‐course experiments were performed for 24, 48, and 72 h. The results revealed that cell proliferation decreased in a time‐dependent manner in both the cell lines in response to siRNA‐mediated knockdown (**Figure** **2**A) and treatment with 5 µm TST (Figure 2B). These results highlight the crucial role of FOXM1 in regulating cell proliferation and confirm the robustness of siRNA‐mediated knockdown and TST‐mediated inhibition of FOXM1 expression in lung cancer cells. siFOXM1‐treated cells or TST‐treated cells were analyzed for DNA content using flow cytometry. Consistent with our previous observations, FOXM1 knockdown by siFOXM1 (Figure 2C) or inhibition by TST (Figure 2D) increased the sub G0/G1 population and decreased the S phase population in a time‐dependent manner compared to cells transfected with negative control (NC) siRNA or dimethyl sulfoxide (DMSO) treatment. Moreover, both cell lines treated with TST displayed an increased G2‐phase population at the expense of the S‐phase population when compared to those treated with DMSO. These results confirm that FOXM1 depletion alters cell cycle progression in NSCLC cells and is important in the G1‐S phase and G2‐M phase transition. In addition, we observed a significant and time‐dependent increase in the apoptotic cell population (annexin V/PI‐positive cells) in FOXM1‐depleted cells following either siRNA (Figure 2E) or TST treatment (Figure 2F).

We further investigated the effect of FOXM1 knockdown on c‐MYC, cyclin B1, cyclin E1, and cyclin D1 in both cell lines by western blot analysis. The protein levels of c‐MYC, cyclin B1, and cyclin D1 significantly decreased following FOXM1 knockdown by siRNA (Figure 2G) and TST treatment (Figure 2H). The collective findings indicate that the FOXM1 inhibitor TST could be a promising candidate for targeting the proliferation and survival of NSCLC cells, as reported previously in other studies.

### 2.3 FOXM1 Depletion Results in PD‐L1 Downregulation in Cell Membranes of Lung Cancer Cells

**Figure 3:**
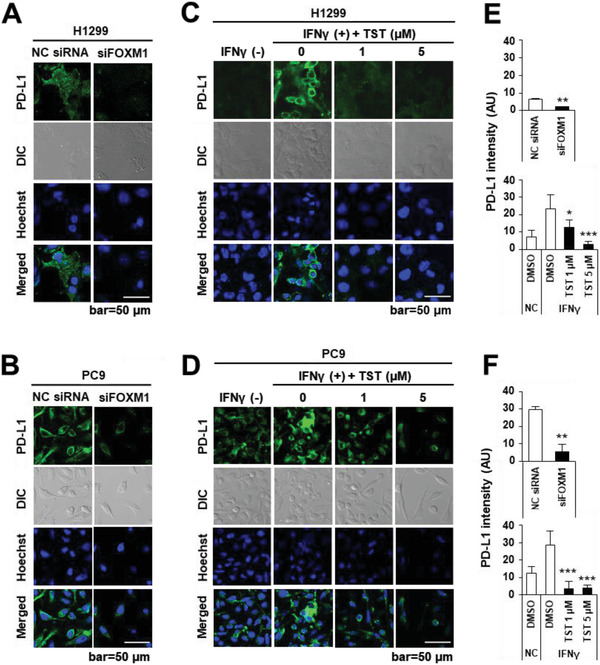
TST‐mediated downregulation of FOXM1 is associated with reduced PD‐L1 expression on the cell membrane. Representative confocal images of A) H1299 and B) PC9 cells stained for FOXM1 or PD‐L1 proteins upon transfection with siFOXM1 or NC siRNA for 72 h. Hoechst 33342 was used to stain nuclei. PD‐L1 intensity quantification histogram for C) H1299 and D) PC9 cells stained for FOXM1 or PD‐L1 proteins treated with TST at a concentration of 1 or 5 µm in the presence of IFN*γ* (20 ng mL^−1^) for 48 h. PD‐L1 intensity quantification histogram for E) H1299 and F) PC9 cell line. Error bars represent standard error of the mean (SEM) *p < 0.05, **p < 0.01, ***p < 0.001.

Since PD‐L1 is a transmembrane protein, and FOXM1 knockdown by siRNA or TST treatment reduces total PD‐L1 protein levels, we performed immunocytochemistry (ICC) to evaluate whether this effect was phenotypically observed in the cell membrane. Membrane PD‐L1 enrichment was also evaluated in H1299 and PC9 cells by ICC after transfection with siFOXM1 or NC siRNA for 72 h. PD‐L1 membrane expression in H1299 control cells was significantly higher than that in siFOXM1‐transfected cells (**Figure** **3**A). FOXM1 downregulation resulted in a 67.4% reduction in PD‐L1 expression (Figure 3E). siFOXM1‐transfected PC9 cells downregulated FOXM1 and this resulted in an 81.68% reduction in PD‐L1 intensity (Figure 3B,F).

Given the compelling evidence that TST treatment reduces overall PD‐L1 protein levels in lung cancer cells in the presence of interferon‐gamma (IFN*γ*), we investigated the effect of TST on PD‐L1 enrichment in the cell membrane. IFN*γ*‐boosted (IFN*γ*(+)) membrane PD‐L1 levels were observed in both cell lines (Figure 3C,D). PD‐L1 reduction was evident in the membranes of TST‐treated H1299 cells (Figure 3C) and PC9 cells (Figure 3D). FOXM1 downregulation resulted in 87.23% and 85.82% reduction in PD‐L1 intensity in H1299 (Figure 3E) and PC9 (Figure 3F) cells, respectively, compared to that in the DMSO control. These findings suggest a concomitant association between FOXM1 depletion and decreased membrane PD‐L1 expression.

Collectively, these findings implicate FOXM1 inhibition by TST treatment as an effective strategy to target the expression of intracellular and cell membrane PD‐L1.

### 2.4 FOXM1 Translocation to the Nucleus is Abrogated by TST

**Figure 4:**
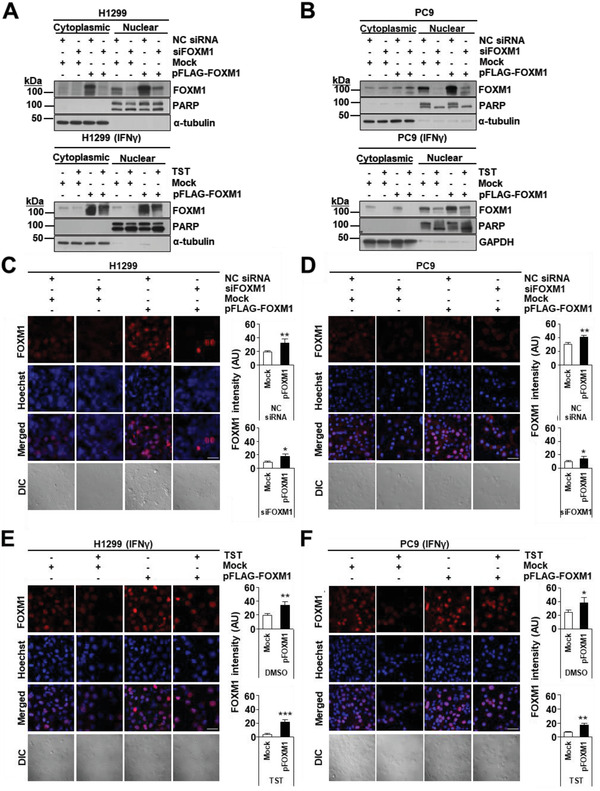
FOXM1 translocation to the nucleus is abrogated by TST treatment. A) H1299 cells were transfected with FOXM1 isoform b (pFLAG‐FOXM1) or an empty vector (Mock) for 48 h. Changes in FOXM1 protein levels following siFOXM1 knockdown for 72 h or TST treatment for 48 h were assessed by western blotting. B) FOXM1 overexpression in PC9 and transfection with FOXM1 isoform b (pFLAG‐FOXM1) or an empty vector (Mock) for 48 h. Changes in FOXM1 protein levels following siFOXM1 knockdown for 72 h or TST (5 µm) treatment for 48 h were assessed by western blotting. C) H1299 cells were transfected with FOXM1 isoform b (pFLAG‐FOXM1) or an empty vector (Mock) for 48 h, and siFOXM1 for 72 h or TST for 48 h. D) PC9 cells were transfected with FOXM1 isoform b (pFLAG‐FOXM1) or an empty vector (Mock) for 48 h, and siFOXM1 for 72 h or TST for 48 h. E) H1299 cells that were transfected with FOXM1 isoform b (pFLAG‐FOXM1) or an empty vector (Mock) for 48 h and treated with TST in presence of IFN*γ* (20 ng mL^−1^) for 48 h. F) PC9 cells that were transfected with FOXM1 isoform b (pFLAG‐FOXM1) or an empty vector (Mock) for 48 h and treated with TST in presence of IFN*γ* (20 ng mL^−1^) for 48 h. Confocal images were obtained by staining cells for FOXM1 and by staining nuclei with Hoechst 33342 (C–F). Error bars represent standard error of the mean (SEM) *p < 0.05, **p < 0.01, ***p < 0.001.

The nuclear translocation of FOXM1 has been highlighted as a prominent feature of many cancers. We investigated FOXM1 enrichment in the nuclei of pFLAG‐FOXM1‐transfected H1299 and PC9 cells, followed by siFOXM1 knockdown or TST treatment (5 µm) in the presence of IFN‐*γ* (20 ng mL^−1^). Interestingly, although FOXM1 was present in the nucleus and cytoplasm, overexpression of FOXM1 increased nuclear translocation of cytosolic FOXM1. However, siFOXM1‐mediated FOXM1 inhibition or TST treatment decreased the protein levels and nuclear translocation of FOXM1 in H1299 (**Figure** **4**A) and PC9 cells (Figure 4B). Compared to mock transfection, FOXM1 overexpression recovered FOXM1 intensity by 48.85% and 84.33% for siFOXM1 and TST treatments in H1299 cells, respectively. The recovery values in PC9 cells were 30.95% and 57.56%, for siFOXM1 and TST treatments, respectively. ICC analysis revealed that FOXM1 was predominantly localized in the nuclei of H1299 cells transfected with pFLAG‐FOXM1 (Figure 4C) and PC9 cells (Figure 4D) transfected with NC siRNA, compared to siFOXM1 transfected cells. Similarly, in H1299 cells (Figure 4E) and PC9 cells (Figure 4F) transfected with pFLAG‐FOXM1, FOXM1 was predominantly localized in the nuclei, compared to TST‐treated cells in which FOXM1 expression and translocation to nuclei were abrogated. In pFLAG‐FOXM1‐mediated FOXM1 overexpression was significantly reduced to 54.22% in H1299 cells (*p* < 0.01) and 35% in PC9 cells (*p* < 0.001) by siRNA‐mediated knockdown. In the TST‐treated cells, FOXM1 overexpression was significantly reduced to 63.7% in H1299 cells (*p* < 0.05) and 44% in PC9 cells (*p* < 0.01).

### 2.5 PD‐L1 Expression is Recovered by FOXM1 Overexpression

**Figure 5:**
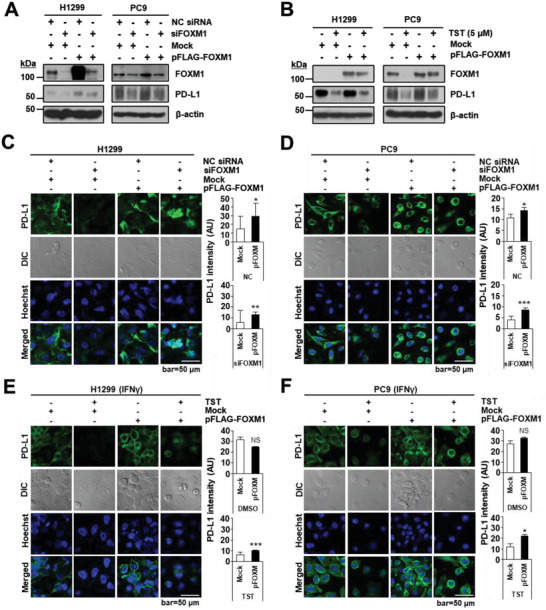
Reduced PD‐L1 expression is recovered by overexpression of FOXM1. A) Cells containing mock or pFLAG‐FOXM1 plasmids were transfected either with NC siRNA or siFOXM1 for 72 h or B) treated with DMSO or TST (5 µm) in the presence of IFN*γ* (20 ng mL^−1^) for 48 h. Immunoblot analysis using indicated antibodies was performed in H1299 and PC9 cells. C) H1299 cells or D) PC9 cells were transfected with FOXM1 isoform b (pFLAG‐FOXM1) or an empty vector (Mock) for 48 h and siFOXM1 for 72 h. Confocal images were obtained by staining cells for FOXM1 or Hoechst 33342 for nuclei. H1299 cells (E) or PC9 cells (F) were transfected with FOXM1 isoform b (pFLAG‐FOXM1) or an empty vector (Mock) for 48 h and treated with TST in presence of IFN*γ* (20 ng mL^−1^) for 48 h. Confocal images were obtained by staining cells for PD‐L1 or Hoechst 33342 for nuclei. Error bars represent standard error of the mean (SEM) *p < 0.05, **p < 0.01, ***p < 0.001.

To support our previous findings, we confirmed the recovery of PD‐L1 expression by inducing the overexpression of FOXM1 in FOXM1‐knockdown cell lines. H1299 and PC9 cells were transfected with NC siRNA or siFOXM1, followed by transfection with pFLAG‐FOXM1 or an empty vector for 72 h. Intriguingly, western blot analysis and confocal imaging of cells revealed that siFOXM1‐induced PD‐L1 downregulation was recovered in pFLAG‐FOXM1‐transfected cells, but not in the mock‐transfected cells. FOXM1 allowed the recovery of PD‐L1 expression on the membranes of H1299 and PC9 cells by 55.9% and 51.8%, respectively (**Figure** **5**A,C,D). Similarly, we examined whether FOXM1 overexpression could lead to the recovery of reduced PD‐L1 expression in H1299 and PC9 cells treated with TST (5 µm) for 48 h in the presence of IFN*γ*. Parallel to the recovery of PD‐L1 expression in FOXM1 knockdown cells, FOXM1 overexpression also rescued PD‐L1 expression in TST‐treated H1299 and PC9 cells. As shown by western blotting (Figure 5B) and confocal microscopy (Figure 5E,F), FOXM1 allowed PD‐L1 expression recovery on the cellular membrane of H1299 and PC9 cells by 38.7% and 46%, respectively. Additionally, the elevated level of PD‐L1 by pFLAG‐FOXM1 overexpression, which mimics the situation in a malignant tumor mass, was significantly reduced to 52.6% in H1299 cells (*p* < 0.05) and 59.2% (*p* < 0.01) in PC9 cells by siRNA, and by 41.9% in H1299 cells (*p* < 0.01) and 68.0% in PC9 cells (*p* < 0.05) by TST treatment. Collectively, these results further support the hypothesis that PD‐L1 is a downstream target of FOXM1 and is positively regulated by FOXM1, implying that PD‐L1 levels can be tightly regulated by manipulating FOXM1.

### 2.6 FOXM1 Regulates PD‐L1 Expression by Binding Directly to its Promoter

**Figure 6:**
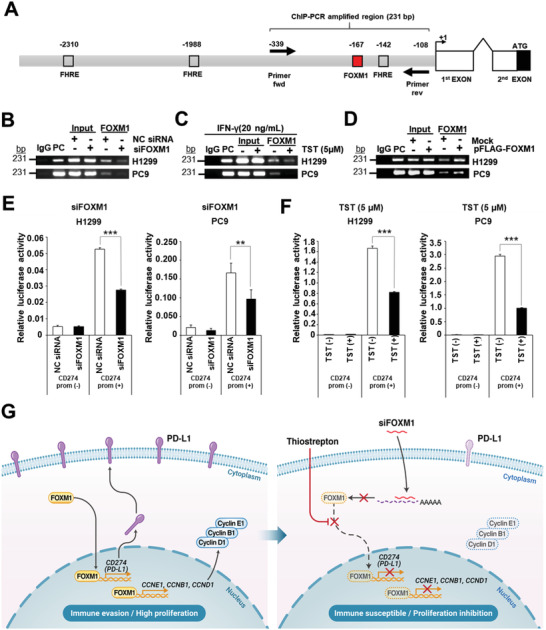
FOXM1 mediates PD‐L1 expression by binding to its promoter A) Chromatin immunoprecipitation (ChIP) analysis of CD274 proximal promoter region. Primers amplifying a 231 bp PCR product including FOXM1 forkhead response element (FHRE) on the CD274 promoter region (−339/−108 bp) were designed. H1299 and PC9 cells were either transfected with B) siFOXM1 or C) treated with TST in presence of IFN*γ* (20 ng mL^−1^) for 48 h or D) transfected with pFLAG‐FOXM1 or an empty vector (Mock) for 48 h. Pellets were used for ChIP assays using an anti‐FOXM1 antibody or IgG antibody as a negative control. The coimmunoprecipitated DNA was amplified by semi‐quantitative PCR. H1299 and PC9 cells were transiently transfected with pGL3‐CD274 promoter 2 kb luciferase vector that includes a 2 kb promoter region of the CD274 gene. Luciferase activity was measured from cell lysates treated with siRNA E) for 72 h or F) TST (5 μM) in the presence of IFNγ (20 ng mL^1^) for 48 h. CD274 promoter transcription activation was quantified using a dual luciferase assay. In all assays, luciferase activity was normalized to the *Renilla* luciferase activity. Error bars represent standard error of the mean (SEM); *p < 0.05, **p < 0.01, and ***p < 0.001. G) Proposed model of FOXM1‐mediated regulation of PD‐L1.

To investigate the detailed mechanism by which FOXM1 induces PD‐L1 expression, we analyzed ChIP‐seq data from the ENCODE 3 database^[^ ^22^ ^]^ accessed from the UCSC genome browser (Human assembly Dec 2013, GRCh38/hg38). In the K562 human immortalized myelogenous leukemia cell line, FOXM1 enrichment was noted on the promoter region with a peak signal of ≈167 bp upstream of the CD274 transcription start site (red box in **Figure** **6**A and Figure S1, Supporting Information). In addition, the TAAAC FOXM1 consensus DNA‐binding domain (DBD) was detected nearby (≈142 bp)^[^ ^23^ ^]^ as well as in two distant regions (−1988 and −2310 bp) from the CD274 transcription start site (Figure 6A).

Based on these in silico data, we hypothesized that the CD274 promoter region of lung cancer cells is a potential target of the FOXM1 TF. Thus, we performed a chromatin immunoprecipitation‐PCR (ChIP‐PCR) assay by excluding the FOXM1‐CD274 DNA complexes using an anti‐FOXM1 antibody. The CD274 promoter region flanking putative FOXM1‐binding sites (−167 nt) was detected by semi‐quantitative PCR in H1299 and PC9 cells, compared to the dramatic decrease in FOXM1 binding to the CD274 promoter observed in the siFOXM1‐transfected cells (Figure 6B) and the TST‐treated cells (Figure 6C) with their respective controls. pFLAG‐FOXM1‐transfected cells exhibited increased binding of FOXM1 to the CD274 promoter compared with mock‐transfected cells (Figure 6D). These results indicate that FOXM1 binds directly to the CD274 promoter. Thus, FOXM1 directly regulated PD‐L1 transcription by binding to its promoter. Targeting FOXM1 abrogates the FOXM1‐mediated regulation of PD‐L1 expression.

To further support our findings, we performed a dual‐luciferase reporter assay using a CD274 promoter‐luciferase construct to determine whether depletion of FOXM1 binding by siRNA or TST treatment significantly decreases CD274 transcriptional activity. Consistent with the ChIP‐PCR results, the depletion of FOXM1 by siRNA significantly reduced the relative luciferase activity by 40% in H1299 cells (*p* < 0.001) and by 42% in PC9 cells (*p* < 0.01) (Figure 6E). Correspondingly, inhibition of FOXM1 by TST in the presence of IFN*γ* also significantly decreased CD274 promoter activity, as indicated by the decrease in relative luciferase activity by 53% in H1299 cells (*p* < 0.001) and by 66% in PC9 cells (*p* < 0.001) (Figure 6F).

These collective findings support the conclusion that FOXM1 translocation to the nucleus positively regulates PD‐L1 expression by directly binding to the CD274 promoter in both H1299 and PC9 cell lines. A new model for FOXM1‐mediated regulation of PD‐L1 expression in lung cancer was proposed. In this model, FOXM1 regulated PD‐L1 expression by directly binding to the CD274 promoter, leading to PD‐L1 transactivation (Figure 6G). PD‐L1 upregulation induced by FOXM1 results in immune evasion of lung tumor cells. Hence, targeting FOXM1 using TST, a natural FOXM1 inhibitor that appears to abrogate immune evasion and reduce tumor growth, is a new potential treatment strategy for PD‐L1‐mediated immune evasion in lung cancer cells.

### 2.7 FOXM1 Inhibition Regulates Tumor Growth and PD‐L1 Expression In Vivo

**Figure 7:**
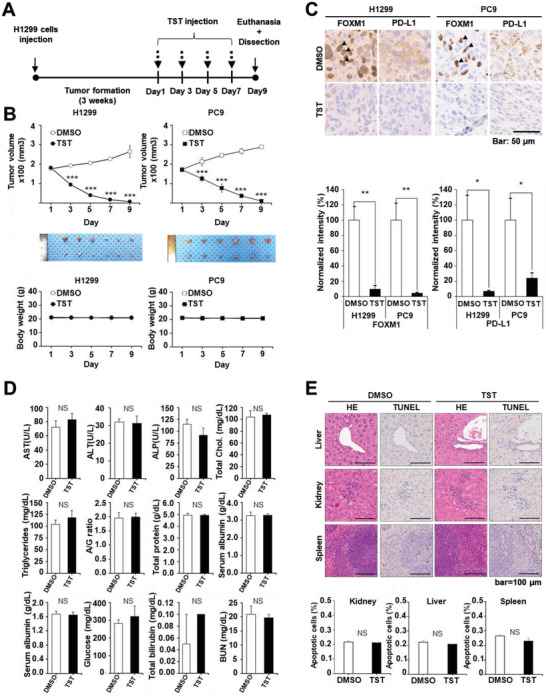
TST abrogates tumor growth and PD‐L1 expression in vivo. A) Timeline of in vivo experiments performed on BALB/c mice. H1299 cells (5 × 10^6^) were injected intraperitoneally and left for 3 weeks to form tumors. On day 21, mice were injected with TST at a dose of 17 mg kg^−1^ at indicated time points represented by black arrows. B) Top: Tumor growth curves of the mice (*n* = 6 per group). Middle: Tumor masses after sacrifice. Bottom: Body weight change. C) Immunohistochemistry of FOXM1 and PD‐L1 of tumor tissues (scale bar, 50 µm). Error bars represent standard error of the mean (SEM), NS = non‐significant, *p < 0.05, **p < 0.01, ***p <0.001. D) Blood biochemical data of the mice in the DMSO‐ and TST‐treated groups on day 9 (*n* = 4). Alanine aminotransferase (ALT), aspartate aminotransferase (AST), alkaline phosphatase (ALP), glucose (Glu), blood urea nitrogen (BUN), creatinine (Crea), total bilirubin (T‐Bill), total cholesterol (T‐chol), triglycerides (TG), total protein (TP), serum albumin (Alb), serum globulin (Glo), and serum albumin/serum globulin ratio (A/G ratio) were analyzed. E) Representative images of H&E‐ and TUNEL‐stained tissue sections of major organs were collected from DMSO and TST treatment groups on day 9 post‐TST injection.

To further support the in vitro findings, the effect of the FOXM1 inhibitor, TST, on tumor growth and PD‐L1 expression was examined in vivo. Human H1299 or PC9 cells (5 × 10^6^) were subcutaneously implanted into BALB/c nude mice (*n =* 6 per group). When the average size of the xenografted tumor masses reached ≈170 mm^3^, TST was intraperitoneally injected into the mice at a dose of 17 mg kg^−1[^ ^24^ ^]^ four times every 2 days (**Figure** **7**A). Regular monitoring of tumor volume during inhibitor treatment revealed a significant decrease in tumor volume in the TST‐treated group compared with that in the control group. No difference in body weight was observed between the two groups after 9 days (Figure 7B). 2 days after the final TST injection, the mice were sacrificed. Tumor tissues were collected, sectioned, and stained to analyze the changes in FOXM1 and PD‐L1 expression levels. Immunohistochemical analyses demonstrated that FOXM1 and PD‐L1 were highly expressed in the lung tumors of control mice, but were significantly downregulated in TST‐treated tumors (Figure 7C). FOXM1 was primarily localized in the nuclei of control tumor cells but exhibited faint immunopositivity in the nuclei of TST‐treated tumor cells. These findings indicated that, in accordance with FOXM1 expression, PD‐L1 was positively expressed in control tumors of H1299 and PC9 xenografts. In contrast, PD‐L1 expression was significantly decreased by TST treatment (Figure 7C). Thus, it can be concluded that FOXM1 inhibition is critical for the regulation of PD‐L1 expression.

Serum biochemical analysis was performed to further evaluate the potential toxicity of TST in vivo. The levels of blood biomarkers were not significantly changed in the TST‐treated groups compared to those in the DMSO control (Figure 7D and Table S2, Supporting Information). In addition, no significant histological changes in vital organs were observed in the TST‐treated group, as demonstrated by hematoxylin and eosin (H&E) staining (Figure 7E). Terminal deoxynucleotidyl transferase dUTP nick end labeling (TUNEL) analysis of the tissue sections corroborated that TST treatment has no effect on apoptotic cell death in normal tissues when compared to DMSO control tissues (Figure 7E).

Collectively, these findings suggest that TST‐mediated FOXM1 inhibition could abrogate immune evasion and effectively reduce tumor growth.

### 2.8 FOXM1 Inhibition and Anti‐4‐1BB Blockade Resulted in Synergistic Antitumor Effect in Syngeneic Murine LLC‐1 Lung Carcinoma Model In Vivo

**Figure 8:**
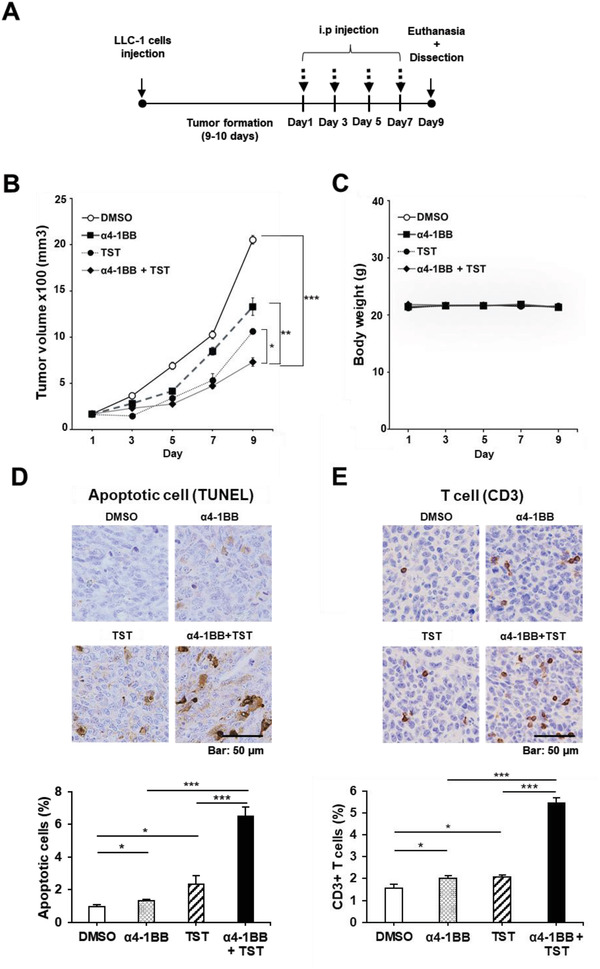
Synergistic anti‐tumor effect of TST and anti–4‐1BB combination in syngeneic Lewis lung carcinoma (LLC‐1) model in vivo. A) Timeline of in vivo experiments performed on C57BL/6 mice (*n* = 6). B) The size of tumors was measured on indicated days. C) The bodyweight of mice was measured on indicated days for 9 days post‐TST and/or anti‐4‐1BB injection. D) Representative images of TUNEL‐stained tissue sections of collected tumor masses from DMSO, TST, anti–4‐1BB, or TST+anti–4‐1BB treatment groups on day 9 post‐treatment (scale bar, 50 µm). E) Immunohistochemistry of CD3^+^ T‐cells of tumor tissues (scale bar, 50 µm). All results are expressed as mean ± SEM. NS = non‐significant, *p < 0.05, **p <0.01, ***p < 0.001.

We extended our study to evaluate the therapeutic potential of TST in cancer immunotherapy using an LLC‐1 syngeneic tumor model (**Figure** **8**A). It is reported that LLC‐1 tumors are resistant to ICI treatment.^[^ ^25^ ^]^ Lewis lung carcinoma LLC‐1 murine lung cancer cells (5 × 10^5^) were injected subcutaneously into C57BL/6N mice (*n =* 6 per group) and allowed to form tumors with a volume of 150 mm^3^ for 10 days. The mice were injected with TST at a dose of 17 mg kg^−1^ and anti‐4‐1BB antibody at a dose of 10 mg kg^−1^ at indicated time points represented by black arrows. The injection dose of an anti‐4‐1BB antibody was chosen based on the finding of a prior study that demonstrated its treatment efficacy in the E. G7 tumor‐implanted C57BL/6 mouse model.^[^ ^26^ ^]^ Treatment of the tumor‐bearing mice with anti–4‐1BB or TST alone resulted in 38% and 48% inhibition of tumor growth, respectively, compared to that in the control group (Figure 8B). Notably, combined treatment with anti–4‐1BB and TST induced significantly enhanced therapeutic outcomes in tumor growth inhibition, recording a 65% of tumor growth inhibition in the tumor‐bearing mice relative to the control group (*p* < 0.001). No differences in body weight were observed between the experimental groups after day 9 (Figure 8C).

Next, TUNEL staining of tumor sections on day 9 was performed to investigate apoptotic cell death in the tumors (Figure 8D). The number of apoptotic tumor cells was 1.38 and 2.46‐folds higher in the anti‐4‐1BB‐treated (*p* < 0.05) and TST‐treated groups (*p* < 0.05), respectively, than in the control group. As expected, the combination of TST with anti‐4‐1BB synergistically increased the number of apoptotic LLC‐1 tumor cells by 4.92‐folds and 2.76‐folds (CDI = 0.5) compared to the anti‐4‐1BB‐treated group (*p* <0.001) or TST‐treated group (*p* <0.001), respectively (Figure 8D).

Furthermore, immunohistochemical staining for CD3 in tumor sections revealed a 1.28‐ and 1.32‐fold increase in CD3^+^ T cells in the anti‐4‐1BB‐treated (*p* < 0.05) and TST‐treated (*p* < 0.05) tumors, respectively (Figure 8D), compared to the control group. Interestingly, the combination of TST with anti–4‐1BB also increased the CD3^+^ T‐cell frequency in the tumor (CDI = 0.49) by 2.72‐folds and 2.63‐folds compared to the anti‐4‐1BB treated group (*p* <0.001) or TST‐treated group (*p* <0.001), respectively (Figure 8E). These results indicate that combined TST and anti–4‐1BB treatment has a significant synergistic effect on the pro‐immunogenic and antitumor activity.

## 3 Discussion

Lung cancer is the most common type of cancer and has strong medical importance, owing to the ever‐increasing mortality associated with this cancer every year.^[^ ^27^ ^]^ Anti‐PD‐L1 therapy is a novel strategy for targeting immune checkpoints. However, the complex regulatory mechanisms involved are not fully understood.

In the present study, we addressed the relationship between FOXM1, an oncogenic TF, and PD‐L1, an immune checkpoint protein, in H1299 and PC9 NSCLC cells. We evaluated the expression levels of FOXM1 and PD‐L1 levels on a tumor tissue sample from a patient with LUAD by tissue staining and overall survival analysis. We observed that simultaneous alterations in the expression levels of FOXM1 and PD‐L1 correlated with a poor prognosis (Figure 1A,B), thus we hypothesized that FOXM1 is a putative regulator of PD‐L1. Our results provide conclusive evidence to support our initial hypothesis of this mechanism. We also highlighted the potency of TST, a FOXM1 inhibitor, in reducing the proliferation of lung cancer cells and the expression of PD‐L1 through FOXM1 inhibition.

Several studies have shown that PD‐L1 plays a critical role in immune escape by providing interaction between tumor cells and cytotoxic T lymphocytes within the tumor microenvironment.^[^ ^28^ ^]^ PD‐L1 expression is an important biomarker for predicting response to cancer therapy,^[^ ^29^ ^]^ and is also a valuable target for ICIs in cancer treatment.^[^ ^30^ ^]^ We investigated the effect of FOXM1 on PD‐L1 expression, considering that FOXM1 is a promising target for treating many cancers, and the control of its aberrant expression, as a result of mutations, in tumor cells is a hallmark of progression and tumorigenesis of NSCLC.^[^ ^31^ ^]^ In particular, the correlations between FOXM1 and cell cycle‐related proteins as well as the prognosis in lung cancer patients are reported by Liang and Kim et al.^[^ ^32^, ^33^ ^]^

In accordance with the clinical data (Figure 1A,B) and transcriptome profiling (Figure 1D), FOXM1 regulated PD‐L1 expression both transcriptionally (Figure 1F–H) and at the protein level (Figure 1I–K). Considering that PD‐L1 expression is induced by intrinsic oncogenic signaling via phosphoinositide 3‐kinase^[^ ^34^ ^]^ and in response to extrinsic stimuli activating IFN*γ* or NF‐*κ*B signaling,^[^ ^35^ ^]^ treatment of FOXM1 depletion using siRNA (siFOXM1) or TST, leads to a decrease in PD‐L1 transcription, as demonstrated by semi‐quantitative PCR (Figure 1E,G) and RNA‐seq (Figure 1C) analysis. Additionally, overexpression of FOXM1 in lung cancer cells increased PD‐L1 expression in FOXM1‐depleted cells (Figure 1F), further supporting the hypothesis that FOXM1 is a positive regulator of PD‐L1 expression. Efficient reduction of PD‐L1 expression could be achieved by inhibiting FOXM1 protein via siRNA‐mediated knockdown or TST treatment under conditions that mimic malignant tumors (Figures 3, 4, and 5).

ChIP‐PCR and luciferase assays provided direct evidence of FOXM1 binding to DNA within the proximal promoter region of the CD274 gene and subsequently activated transcription (Figure 6B–D). These findings highlight the direct role of FOXM1 in modulating PD‐L1 at the transcriptional level and suggest that the PD‐L1 promoter is crucial for FOXM1‐mediated regulation of PD‐L1 expression.

FOXM1 knockdown reduced PD‐L1 expression, induced cell death (Figure 2C,D), and downregulated other proliferation‐associated proteins, including cyclin D1, cyclin E1, cyclin B1, and MYC (Figure 2G,H). More significantly, compared to the use of a much higher TST dose (500 mg kg^−1^),^[^ ^36^ ^]^ our in vivo xenograft tumor study using a low TST dose (17 mg kg^−1^) resulted in significant tumor shrinkage (Figure 7B) without any apparent side effects on normal tissues (Figure 7D,E and Table S2, Supporting Information). A significant decrease in PD‐L1 levels was observed (Figure 7C), revealing the dual role of FOXM1 in regulating the proliferation as well as the immune evasion of lung cancer cells (Figure 8). As mentioned above, it has been reported that the effect of ICIs on tumor treatment is not significant for large or rapidly growing tumors.^[^ ^37^ ^]^ Therefore, a significant reduction in tumor volume by the FOXM1 inhibitor, in addition to its effect on PD‐L1 expression, could be an important factor in enhancing the therapeutic outcome of immunotherapy in lung cancer. Interestingly, TST treatment in a murine syngeneic model induced tumor shrinkage through TST‐induced programmed cell death (Figure 8B,D) as well as a significant increase in CD3^+^ T cells at the tumor site (Figure 8E), a feature of a strong immune response to the tumor (Figure 8E)^[^ ^38^ ^]^ without any significant change in body weight (Figure 8C). Notably, the combination of TST and anti‐4‐1BB significantly enhanced the retardation of tumor growth and attracted T‐cells to the tumor region in the immune‐resistant LLC1 lung tumor model. It should be also noted that 78% of tumor growth inhibition could be obtained in the combined TST and anti‐4‐1BB treatment group (CDI = 0.94) when treatment was started at the smaller tumor size (i.e., ≈80 mm^3^) (Figure S3, Supporting Information). These results suggest that the application of TST is a clinically meaningful strategy to suppress tumor growth and enhance the efficacy of immune therapy when combined with immune checkpoint inhibitors.

In our previous study, we also confirmed that other FOXM1 inhibitors, such as RCM‐1 and FDI‐6, also reduced PD‐L1 expression in vitro, even though a dose more than four times higher than TST was needed to obtain similar effects (data not shown). These results indicated the potential of FOXM1 inhibitors as novel drug candidates for regulating PD‐L1 expression.

Our findings highlight the immunoregulatory role of FOXM1 in cancer cells, suggesting the need for future investigations exploring FOXM1 as a prognostic biomarker, in addition to PD‐L1, for cancer immunotherapy. The collective findings of this study suggest the potential value of FOXM1 in the inhibition of immune checkpoint molecules alone or in combination with other conventional immune checkpoint inhibitors and may prove to be a better strategy for improving the overall prognosis of patients with NSCLC.

## 4 Experimental Section

### Datasets for Informatic Analyses of FOXM1 and PD‐L1 in LUAD

To assess the survival of PD‐L1 and FOXM1, and to determine the role of FOXM1 and PD‐L1 in the prognosis of LUAD, TCGA LUAD Firehose Legacy (*n =* 586) available on cBioPortal for cancer genomics open access resources was used.^[^ ^39^ ^]^

### Cell Culture

H1299 and PC9 cells purchased from ATCC (Manassas, VA, USA) were cultured at 37 °C and 5% CO_2_ in RPMI‐1640 medium (#10–040‐CV; Corning, New York, NY, USA) supplemented with 10% fetal bovine serum (#35–010‐CV; Corning) and a penicillin–streptomycin antibiotic cocktail (#15240062; Thermo Fisher Scientific, Waltham, MA, USA).

### siRNA and Plasmid Transfection

H1299 and PC9 LUAD cells were transfected with 5 nM negative control (NC) or FOXM1 siRNA (siFOXM1) (QIAGEN, Hilden, Germany) using Lipofectamine RNAiMAX (#13778150; Thermo Fisher Scientific) (Figure S2, Supporting Information). The siRNA sequences are listed in Table S3, Supporting Information. Cells that underwent reverse transfection were maintained at 37 °C and 5% CO_2_ and collected at the end of each experiment.

Human FOXM1 isoform b (pFLAG‐FOXM1)‐expressing plasmid or empty vector (pFLAG‐CMV2; Mock) was transfected into H1299 and PC9 cells using Lipofectamine 2000 reagent (#11668019; Thermo Fisher Scientific) according to the manufacturer's instructions.

### Reverse Transcription and Semi‐Quantitative PCR

Total RNA was extracted and purified using an RNeasy kit (#74004; QIAGEN) according to the manufacturer's protocol. mRNA reverse transcription was done using the PrimeScript 1^st^ Strand cDNA Synthesis Kit (#6110A; TaKaRa Bio, Otsu, Japan). Quantitative PCR was performed using primers specific for the CD274 and FOXM1 genes. Each pair of primers was designed using Primer 3 software.^[^ ^40^ ^]^ The *β*‐actin gene was used as an internal control to normalize target gene expression. Primer pair sequences are listed in Table S4, Supporting Information.

### Geneset Enrichment Analysis

DEGs in response to siFOXM1 knockdown were cataloged. Significantly downregulated genes (lower than 0.5‐fold between NC siRNA and siFOXM1 knockdown H1299 and PC9 cells) (Table S1, Supporting Information) were subjected to GSEA using the Enrichr database.

### Cell Proliferation Assay

H1299 and PC9 cells were grown in a 96‐well plate and treated with 5 µM TST or transfected with siFOXM1 at a density of 3000 cells/well in 6‐well plates. Cell proliferation was recorded at designated times by adding 4‐([3‐(4‐iodophenyl)‐2‐(4‐nitro‐phenyl)‐2H‐5‐tetrazolio]‐1,3‐benzene sulfonate) (WST‐1, #MK400, TaKaRa Bio) for 2 h at 37 °C. The absorbance was measured at 450 nm. All experiments were performed in quadruplicate.

### Cell Cycle Distribution Analysis

Both H1299 and PC9 cells were enzymatically dissociated using TrypLE (#12605‐010; Thermo Fisher Scientific), collected by centrifugation at 2000 × *g* for 2 min, resuspended in phosphate‐buffered saline (PBS), fixed in 70% ethanol, and stored at 4 °C overnight. Fixed cells were pelleted, washed twice with PBS, resuspended in FxCycle PI (propidium iodide)/RNase staining solution (#F10797; Thermo Fisher Scientific), and incubated in the dark at 37 °C for 30 min. Flow cytometry was performed using a FACS‐LSR Fortessa flow cytometer (BD Biosciences, Santa Clara, CA, USA).

### Apoptotic Cell Death Analysis (Annexin V/PI staining)

H1299 and PC9 cells (1 × 10^5^) were cultured in six‐well plates and transfected with siFOXM1 or NC siRNA, or treated with 5 µM TST or DMSO. The fluorescein isothiocyanate Annexin V Apoptosis Detection Kit I was used to stain the cells (#556547; BD Biosciences). The cells were analyzed within 1 h to reduce any adverse effects from staining. Flow cytometry was performed using a FACSVerse Cell Analyzer (BD Biosciences).

### Nuclear Cytosolic Fractionation

To determine protein subcellular localization, lysis of H1299 and PC9 cell pellets was performed for 10 min with Buffer A (10 mM HEPES, 10 mm KCl, 1.5 mM MgCl_2_, 0.5 mM dithiothreitol [DTT], and 0.05% IGEPAL CA‐630 [pH 7.9]). The nuclear fraction was collected by centrifugation of the buffer A lysate at 1000 × *g* for 10 min at 4 °C. After removing the cytoplasmic fraction, Buffer B containing 5 mM HEPES, 0.2 mM EDTA, 1.5 mM MgCl_2_, 0.5 mM DTT, and 26% glycerol v/v (pH 7.9) was applied to the nuclear fraction, and lysis was performed for 30 min on ice.

### Immunoblotting Analysis of Protein Expression

Cells were lysed in RIPA buffer supplemented with a complete mini protease inhibitor cocktail (#04693124001; Roche, Basel, Switzerland) and kept on ice for 30 min. Equal amounts of protein were loaded evenly and resolved by sodium dodecyl sulfate‐polyacrylamide gel electrophoresis and transferred to a polyvinylidene difluoride membrane (#IPVH 00010; Millipore, Billerica, MA, USA) for 1 h at 100 V. Specific primary antibodies against FOXM1 (#sc‐271746), cyclin E1 (#sc‐481), *α*‐Tubulin (#sc‐8035), and *β*‐actin (#sc‐47778) (all from Santa Cruz Biotechnology, Dallas, TX, USA); PD‐L1(#13684S), cyclin D1 (#2978S), cyclin B1(#4138S), c‐MYC (#5605S), and poly (ADP‐ribose) polymerase (PARP, #9542S; Cell Signaling Technology, Beverly, MA, USA) were used to probe for the protein of interest. Horseradish peroxidase‐conjugated secondary antibodies specific for the primary antibodies were used to detect proteins using a pico‐enhanced chemiluminescence substrate (#34577; Thermo Fisher Scientific).

### ICC

The cells were fixed in 4% paraformaldehyde (#30525–89–4; FUJIFILM Wako Chemical, Wako, Japan) for 15 min and blocked with 1% bovine serum albumin in PBS‐00.1% Tween‐20 (PBS‐T) to reduce non‐specific antibody binding. The cells were then incubated overnight with anti‐PD‐L1 antibody (#14598382; eBioscience, San Diego, CA, USA) diluted 1:200, and anti‐FOXM1 antibody (#sc‐271746; Santa Cruz Biotechnology) diluted 1:50 at 4 °C, followed by incubation for 1 h with Alexa Fluor 488‐labeled secondary antibody (#A11029; Thermo Fisher Scientific). Nuclei were stained with Hoechst 33342. After incubation, the cells were washed with PBS and mounted on glass slides using Prolong Glass Antifade mounting medium (#P36982; Thermo Fisher Scientific). Confocal microscopy was performed using a Zeiss 730 Meta microscope (Carl Zeiss, Oberkochen, Germany) and analyzed using Axiovision software (Carl Zeiss).

### ChIP

To cross‐link proteins to DNA, H1299 and PC9 cells were treated for 10 min at 37 °C with 0.7% formaldehyde. Cross‐linking was quenched by adding glycine to the culture medium at a final concentration of (0.125 M) for 5 min. The cells were sonicated using a Bioruptor Next Gen sonicator (Diagenode, Denville, NJ, USA) for 5 min in 30 s on/30 s off cycles to obtain ≈500 bp DNA fragments. Sonicated cell lysates containing genomic DNA fragments were precleared to eliminate unwanted non‐specific components. ChIP was performed using antibodies against FOXM1 (#sc‐271746, Santa Cruz Biotechnology) with rotation at 4 °C overnight, followed by various washing steps, decrosslinking, and digestion of proteins with proteinase K (#P2308; Sigma‐Aldrich, St. Louis, MO, USA). The immunoprecipitated DNA fragments were purified using the phenol–chloroform method. The purified DNA pellets were resuspended in TE buffer (pH 8.0) and used for PCR. Primers specifically detecting the FOXM1 binding region were designed using the Primer3 tool for ChIP‐PCR (Table S4, Supporting Information).

### Dual‐Luciferase Assay

The pGL3 plasmid containing a 2 kb promoter of CD274 (#107003; Addgene, Watertown, MA, USA) was a gift from the Julian Downward lab.^[^ ^41^ ^]^ H1299 and PC9 cells were co‐transfected with the pGL3 2 kb promoter. Cells containing CD274 or the control pGL3‐basic plasmid and the pGL4.70 hRluc *Renilla* luciferase vector (Promega, Madison, WI, USA) were transfected with siFOXM1 or treated with TST in the presence of IFN*γ*. Cells were harvested 48 h post‐transfection, and a dual luciferase assay was performed according to the manufacturer's protocol (#E1910; Promega). At least four independent biological replicates were examined to obtain reliable results.

### Xenograft Tumor Model

All animal studies were reviewed and approved by the Institutional Animal Care and Use Committee of the National Cancer Center Research Institute (NCC‐21–619). H1299 and PC9 (5 × 10^6^) cells were subcutaneously implanted into BALB/c nude mice (CAnN.CgFoxn1nu/CrljOri; Orient Bio, Seoul, Korea). 20 days post‐implantation, mice in the control group (*n =* 6) were intraperitoneally injected with DMSO solution. TST dissolved in DMSO was injected intraperitoneally at a dose of 17 mg kg^−1^ into mice every 2 days before euthanization and sacrifice (*n =* 6). The tumor volume and body weight of the mice were measured every other day. The tumor volume (*V*) was calculated as AB^2^/2, where A and B are long and short diameters (mm), respectively. Two days after the final TST injection, all mice were sacrificed, and tumor tissues were collected for immunohistochemistry (IHC) staining of FOXM1 and PD‐L1. The tissues were fixed in 10% neutralized formaldehyde and embedded in paraffin blocks. Tissue microarray (TMA) slides were made with a 4 mm diameter per sample. TMA slides were sectioned at 4 µm thickness for IHC with anti‐FOXM1 (#20459S) or anti‐PD‐L1 (#13684S) (Cell Signaling Technology) antibodies. Autostainer Link48 (Dako, Agilent Technologies, Santa Clara, CA, USA) was used for analysis. IHC staining was performed using anti‐rabbit‐HRP antibody (K4003, EnVision+ HRP Labelled Polymer Anti‐Rabbit, Dako, Agilent Technologies, Carpinteria, CA, USA) and 3,3'‐diaminobenzidine (DAB) (K3468, DAB+ Chromogen, Dako, Agilent Technologies, Santa Clara, CA, USA) as a substrate, and the sections were counter‐stained with hematoxylin (K8008, EnVision FLEX Hematoxylin, Dako, Agilent Technologies Singapore, Singapore).

To analyze the in vivo toxicity of the tested drugs, four xenograft mice from each group were used for serum biochemical analysis (Table S2, Supporting Information). On day 9, serum samples were collected from the mice in the control and TST‐treated groups. In addition, the vital organs (liver, spleen, and kidney) were collected from mice, sectioned, and stained with H&E to monitor histopathological changes. TUNEL assay (ApopTag Plus Proxidase In Situ kit, EMD Milipore, Temecula, CA, USA) was also performed on the sectioned tissues to analyze apoptotic cell death in response to the tested drug.

### Syngeneic Tumor Model Using Murine Lung Cancer Cell LLC‐1

This animal studies were reviewed and approved by the Institutional Animal Care and Use Committee of the National Cancer Center Research Institute (NCC‐22–675B). Lewis lung carcinoma LLC‐1, murine lung cancer cells (5 × 10^5^) were implanted subcutaneously in 5 weeks' aged C57BL/6N mice (*n =* 6 per group) (OrientBio, Seoul, Korea) and left for 10 days to form tumors near 150 mm^3^ of its volume. Then, mice were injected with TST at a dose of 17 mg kg^−1^ and 4‐1BB antibody at a dose of 10 mg kg^−1^ at indicated time points represented by black arrows (Figure 8A). The tumor volume and body weight of the mice were measured every other day. At day 9, all the mice were sacrificed, and tumor tissues were collected, fixed with formalin, embedded in paraffin, and sectioned for IHC. In addition, TUNEL assay was performed on the sectioned tissues to analyze apoptotic cell death in response to TST and 4‐1BB treatment.

Evaluation of CD3^+^ T‐cells in the tumor sections was performed as previously described.^[^ ^42^ ^]^ CD3^+^ T‐cells were detected by anti‐CD3 antibody (ab16669, Abcam, Cambridge, UK) according to manufacturer's manuals. The positive signal intensity of CD3^+^ T‐cells was analyzed by using a computerized image analysis of ImageJ software (LOCI, University of Wisconsin).^[^ ^43^ ^]^ Intensity of CD3^+^ signals was calculated as a relative ratio of total positive area/total surface area. Pathological images were obtained from Aperio VERSA slide scanning system (Leica Biosystems Imaging, Vista, CA, USA). Under 100 × magnification, three independent and intact computerized microscopic fields of each tissue sample were captured by using Aperio Imagescope software (Leica Biosystems Imaging, Vista, CA, USA) and were used for analysis of the signal intensity of CD3^+^ cells in whole microscopic fields.

In vivo synergistic effect between TST and anti‐4‐1BB treated groups was determined by calculating the CDI (Coefficient of drug interaction). Values of CDI < 1, < 0.7, = 1, and > 1 indicate synergy, significant synergy, additivity, and antagonism, respectively.^[^ ^44^ ^]^

### Statistical Analysis

Differences between the groups were evaluated using the *χ* ^2^ test and Student's *t*‐test. Statistical significance was set at *p* < 0.05. The variation was represented by the standard deviation.

## Conflict of Interest

The authors declare no conflict of interest.

## Author Contributions

H.M. is the first author of this work. S‐H.G. and Y.C. contributed to project conceptualization. H.M., J.‐S.L., Y.E.C, and Y.L. contributed to methodology. H.M. performed software analysis. H.M., J.‐S.L., and Y.E.C. contributed to validation. H.M., J.‐S.L., and Y.C. contributed to formal analysis. H.M., J.‐S.L. and Y.C. contributed to investigation. J.‐S.L. and Y.E.C contributed to providing resources. H.M., J.‐S.L., Y.C., and Y.L. contributed to data curation. H.M., Y.E.C., and J.‐S.L. contributed to visualization. S‐H.G. and M.H.K. contributed to supervision. H.M. wrote the original draft of this manuscript. S‐H.G. and M.H.K. contributed to reviewing and editing. S‐H.G. administered the project. S‐H.G. and Y.C. contributed to funding acquisition. All authors reviewed and approved the final manuscript.

## Supporting information

Supporting Information

## Data Availability

The data that support the findings of this study are available in the supporting information of this article.
