# Supplementary material for: FOXM1 Inhibition Enhances the Therapeutic Outcome of Lung Cancer Immunotherapy by Modulating PD‐L1 Expression and Cell Proliferation
Source: Adv Sci (Weinh). 2022 Aug 17;9(29):2202702. doi: 10.1002/advs.202202702 (PMC9561767; doi:10.1002/advs.202202702)
Supplement: Supplementary file 1 — Supporting Information [file ADVS-9-2202702-s001.pdf]

ADVANCED SCIENCE
Open Access

Supporting Information

for *Adv. Sci.*, DOI 10.1002/advs.202202702

FOXM1 Inhibition Enhances the Therapeutic Outcome of Lung Cancer Immunotherapy by Modulating PD-L1 Expression and Cell Proliferation

Hamadi Madhi, Jeon-Soo Lee, Young Eun Choi, Yan Li, Myoung Hee Kim, Yongdoo Choi and Sung-Ho Goh**

Supporting Information

FOXM1 Inhibition Enhances the Therapeutic Outcome of Lung Cancer Immunotherapy by Modulating PD-L1 Expression and Cell Proliferation

Hamadi Madhi, Jeon-Soo Lee, Young Eun Choi, Yan Li, Myoung Hee Kim, Yongdoo Choi and Sung-Ho Goh**

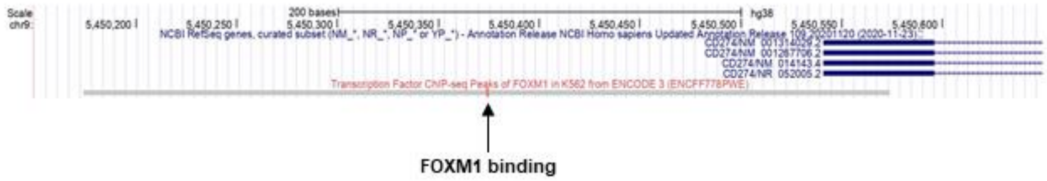

Figure S1. FOXM1 binding to CD274 proximal promoter region. Enrichment of FOXM1 on the promoter region with a peak signal approximately 167 bp upstream of the CD274 transcription start site in the K562 cell line (depicted in red).

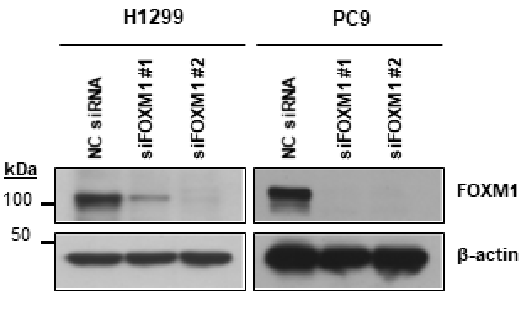

Figure S2. The efficiency of FOXM1 knockdown in H1299 and PC9 cells. Western blot analysis of FOXM1 from cell lysates of H1299 and PC9 cell lines following transfection with NC siRNA or siFOXM1 #1 or siFOXM1 #2 for 72 h.

Syngeneic tumor model using murine lung cancer cell LLC-1: Lewis lung carcinoma LLC-1, murine lung cancer cells (5×10^5) were implanted subcutaneously in 5-weeks aged C57BL/6N mice (*n* = 6 per group) (OrientBio, Seoul, Korea) and left to form tumors near 80 mm^3 of its volume. Then, mice were injected with TST at a dose of 17 mg kg^{-1} and 4-1BB antibody at a dose of 10 mg/kg at the same time points in Figure 8A. The tumor volume and body weight of the mice were measured every other day.

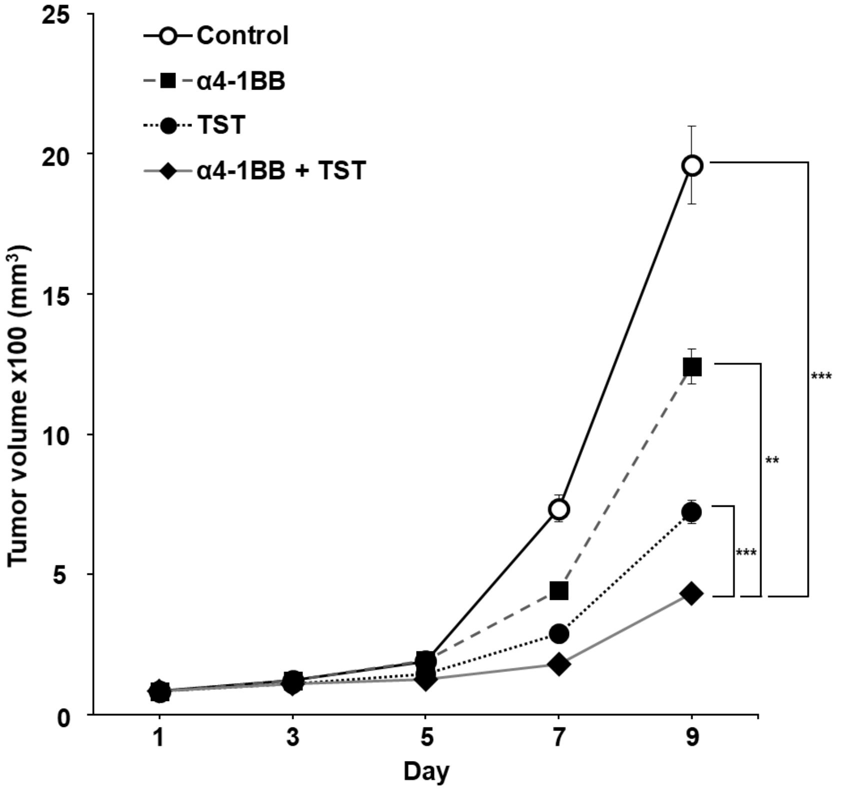

Figure S3. Tumor growth curves of the mice (n = 6 per group) in syngeneic Lewis lung carcinoma (LLC-1) model in vivo. The treatment with anti-4-1BB antibody, TST, and TST plus anti-4-1BB led to inhibition of tumor growth by 36.6%, 63.1%, and 78.0%, respectively, compared to the control group. Synergistic anti-tumor effect could be obtained in the combined TST and anti-4-1BB treatment group compared to the anti-4-1BB and TST treatment alone (CDI = 0.94). Error bars represent standard error of the mean (SEM), NS=non-significant, *p < 0.05, **p < 0.01, **p <0.001.

Table S1. 67 downregulated genes (Fold change < 0.5) by siFOXM1 knockdown in H1299 and PC9.

Gene symbol	Description
ADM	adrenomedullin
ADRB2	adrenoceptor beta 2
AGPAT9	O-acylglycerol-3-phosphate O-acyltransferase 9
AKAP12	A-kinase anchoring protein 12
ATF3	activating transcription factor 3
BHLHE40	basic helix-loop-helix family member e40
CD274	CD274 molecule
CDKN3	cyclin-dependent kinase inhibitor 3
CENPQ	centromere protein Q
CSRNP1	cysteine and serine rich nuclear protein 1
CTGF	connective tissue growth factor
CYR61	cysteine rich angiogenic inducer 61
DKK1	dickkopf WNT signaling pathway inhibitor 1
DUSP1	dual specificity phosphatase 1
DUSP10	dual specificity phosphatase 10
DUSP2	dual specificity phosphatase 2
DUSP5	dual specificity phosphatase 5
EDN1	endothelin 1
EGR1	early growth response 1
EGR3	early growth response 3
EGR4	early growth response 4
ELL2	elongation factor for RNA polymerase II 2
ERRFI1	ERBB receptor feedback inhibitor 1
F2RL1	F2R like trypsin receptor 1
FAM91A1	family with sequence similarity 91 member A1
FOSB	FosB proto-oncogene, AP-1 trancription factor subunit
FOXM1	forkhead box M1
GADD45A	growth arrest and DNA damage inducible alpha
GADD45B	growth arrest and DNA damage inducible beta
GEM	GTP binding protein overexpressed in skeletal muscle
GPR3	G protein-coupled receptor 3
HBEGF	heparin binding EGF like growth factor
IER2	immediate early response 2
IER3	immediate early response 3
JAG1	jagged 1
JUN	Jun proto-oncogene, AP-1 transcription factor subunit
JUNB	JunB proto-oncogene, AP-1 transcription factor subunit
KLF6	Kruppel-like factor 6
KRTAP2-3	keratin associated protein 2-3
MYC	v-myc avian myelocytomatosis viral oncogene homolog
NCEH1	neutral cholesterol ester hydrolase 1
NFKBIZ	NFKB inhibitor zeta

NR4A1	nuclear receptor subfamily 4 group A member 1
NR4A2	nuclear receptor subfamily 4 group A member 2
NR4A3	nuclear receptor subfamily 4 group A member 3
NUAK2	NUAK family kinase 2
PLAU	plasminogen activator, urokinase
PLK2	polo like kinase 2
PTGER4	prostaglandin E receptor 4
RCAN1	regulator of calcineurin 1
RGS2	regulator of G-protein signaling 2
RPL21	ribosomal protein L21
SCARNA16	small Cajal body-specific RNA 16
SGK1	serum/glucocorticoid regulated kinase 1
SNORA61	small nucleolar RNA, H/ACA box 61
SNORA70	small nucleolar RNA, H/ACA box 70
SNORA71C	small nucleolar RNA, H/ACA box 71C
SNORA76C	small nucleolar RNA, H/ACA box 76C
SOX9	SRY-box 9
STC1	stanniocalcin 1
THBS1	thrombospondin 1
TM4SF1	transmembrane 4 L six family member 1
TNFAIP3	TNF alpha induced protein 3
TRIB1	tribbles pseudokinase 1
WNT9A	Wnt family member 9A
ZC3H12A	zinc finger CCCH-type containing 12A
ZFP36	ZFP36 ring finger protein

Table S2. Blood biochemistry test of TST-treated and control Balb/C mice.

	**Control (*n*= 4)**	**TST (*n*= 4)**
**ALT [U/L]**	32.00 ± 1.87	31.25 ± 4.21
**AST [U/L]**	72.00 ± 9.30	82.75 ± 8.58
**ALP [U/L]**	115.00 ± 10.42	91.50 ± 15.04
**Glu [mg/dL]**	281.75 ± 22.86	321.00 ± 58.73
**BUN [mg/dL]**	20.93 ± 2.87	19.70 ± 1.11
**Crea [mg/dL]**	0.03 ± 0.04	0.00 ± 0.00
**T-Bill [mg/dL]**	0.10 ± 0.05	0.10 ± 0.00
**T-chol [mg/dL]**	104.00 ± 10.70	107.50 ± 3.20
**TG [mg/dL]**	103.75 ± 9.07	118.00 ± 14.88
**TP [g/dL]**	4.93 ± 0.18	4.93 ± 0.08
**Alb [g/dL]**	3.25 ± 0.21	3.28 ± 0.08
**Glo [g/dL]**	1.68 ± 0.08	1.65 ± 0.09
**A/G ratio**	1.95 ± 0.19	1.99 ± 0.13

(**Abbreviations:** ALT, alanine aminotransferase; AST, aspartate aminotransferase; ALP, alkaline phosphatase; Glu, glucose; BUN, blood urea nitrogen; Crea, creatinine; T-Bill, total bilirubin; T-chol, total cholesterol; TG, triglycerides; TP, total protein; Alb, serum albumin; Glo, serum globulin; A/G ratio, serum albumin/serum globulin ratio.)

Table S3. The target sequences of siRNAs used in this study.

Name	Target sequence	Supplier
Allstar NC siRNA	Proprietary sequence	Qiagen
siFOXM1 #1	AAC ATC AGA GGA GGA GGA ACC TAA	Qiagen
siFOXM1 #2	TGG GAT CAA GAT TAT TAA CCA	Qiagen

Table S4. Primers used in this study.

Name	Forward sequence	Reverse sequence
β-actin	CAT GTT TGA GAC CTT CAA CAC CCC	GCC ATC TCC TGC TCG AAG TCT AG
FOXM1	ATC TCA GCA CCA CTC CCT TG	CTT GCT GAG GCT GTC ATTCA
CD274	TAT GGT GGT GCC GAC TAC AA	TGC TTG TCC AGA TGA CTT CG
CD274-ChIP	CAA GGT GCG TTC AGA TGT TG	TCC TGA CCT TCG GTG AAA TC
